# Conditional Deletion of EphA4 on Cx3cr1-Expressing Microglia Fails to Influence Histopathological Outcome and Blood Brain Barrier Disruption Following Brain Injury

**DOI:** 10.3389/fnmol.2021.747770

**Published:** 2021-09-24

**Authors:** Eman Soliman, Jatia Mills, Jing Ju, Alexandra M. Kaloss, Erwin Kristobal Gudenschwager Basso, Nathalie Groot, Colin Kelly, Elizabeth A. Kowalski, Mohamed Elhassanny, Michael Chen, Xia Wang, Michelle H. Theus

**Affiliations:** ^1^Department of Biomedical Sciences and Pathobiology, Virginia Tech, Blacksburg, VA, United States; ^2^Department of Pharmacology and Toxicology, Faculty of Pharmacy, Zagazig University, Zagazig, Egypt; ^3^School of Neuroscience, Virginia Tech, Blacksburg, VA, United States; ^4^Center for Engineered Health, Virginia Tech, Blacksburg, VA, United States

**Keywords:** neuroinflammation, TMEM119, peripheral monocytes, Eph signaling, innate immune, traumatic brain injury

## Abstract

Erythropoietin-producing human hepatocellular receptors play a major role in central nervous system injury. Preclinical and clinical studies revealed the upregulation of erythropoietin-producing human hepatocellular A4 (EphA4) receptors in the brain after acute traumatic brain injury. We have previously reported that Cx3cr1-expressing cells in the peri-lesion show high levels of EphA4 after the induction of controlled cortical impact (CCI) injury in mice. Cx3cr1 is a fractalkine receptor expressed on both resident microglia and peripheral-derived macrophages. The current study aimed to determine the role of microglial-specific EphA4 in CCI-induced damage. We used *Cx3cr1*^*CreER*/+^ knock-in/knock-out mice, which express EYFP in Cx3cr1-positive cells to establish microglia, EphA4-deficient mice following 1-month tamoxifen injection. Consistent with our previous findings, induction of CCI in wild-type (WT) *Cx3cr1*^*CreER*/+^*EphA4*^+/+^ mice increased EphA4 expression on EYFP-positive cells in the peri-lesion. To distinguish between peripheral-derived macrophages and resident microglia, we exploited GFP bone marrow-chimeric mice and found that CCI injury increased EphA4 expression in microglia (TMEM119+GFP–) using immunohistochemistry. Using *Cx3cr1*^*CreER*/+^*EphA4* ^*f*/*f*^ (KO) mice, we observed that the EphA4 mRNA transcript was undetected in microglia but remained present in whole blood when compared to WT. Finally, we found no difference in lesion volume or blood-brain barrier (BBB) disruption between WT and KO mice at 3 dpi. Our data demonstrate a nonessential role of microglial EphA4 in the acute histopathological outcome in response to CCI.

## Introduction

Erythropoietin-producing human hepatocellular receptors are the largest family of receptor tyrosine kinase in the CNS. The binding of erythropoietin-producing human hepatocellular (Eph) receptors with their ligands (ephrin), including both classes A and B, initiates bidirectional signaling, which is important in both developing and adult brains (Yang et al., 2018). The role of Eph receptors during brain development includes morphogenesis, neural development, and plasticity (Klein, 2004). In the adult brain, Eph receptor expression is high in different areas, showing structural plasticity, such as the amygdala and hippocampus (Liebl et al., 2003). Following injury, Eph/ephrin signaling is upregulated in a spatiotemporal fashion according to the type and severity of injury (Miranda et al., 1999; Bundesen et al., 2003; Yang et al., 2018). Previous clinical and preclinical studies showed that class A receptor, EphA4, signaling is involved in traumatic brain injury (TBI). EphA4 expression is upregulated in the postmortem brain biopsies of patients who died after acute traumatic brain injury (Frugier et al., 2012). Using the nonhuman primate TBI model, EphA4 was upregulated around the cortical lesion, and this effect was associated with increased neuronal death (Goldshmit and Bourne, 2010). In the murine-controlled cortical impact (CCI) model for TBI, our previous findings demonstrate that EphA4 is overexpressed in injured cortical tissues, and global deficiency of EphA4 showed neuroprotection (Kowalski et al., 2019). Interestingly, conditional EphA4 deficiency in neurons failed to improve a histological outcome after focal TBI in mice (Hanell et al., 2012), indicating the possible role of EphA4 in alternative cell types.

Secondary damage that ensues after the primary injury is attributed to a complex cascade of events that contribute to the neurotoxic milieu that includes blood-brain barrier (BBB) breakdown and influx of peripheral blood-derived components and immune cell recruitment, reactive astrogliosis, and microgliosis. These cells produce reactive oxygen species and inflammatory cytokines resulting in neuronal activity disruption and axonal degeneration, which may progress to apoptosis and neurodegeneration (Ng and Lee, 2019). Microglia, the first line innate immune response in the brain, play a pivotal role in regulating neuroinflammation in TBI (Simon et al., 2017). The role of activated microglia in regulating the tissue microenvironment after TBI is currently under intense investigation, where dual roles in mediating protection or neuronal dysfunction have been established (Loane and Kumar, 2016). The neuroprotective function of microglia is mediated, in part, by the production of trophic factors and their ability to clear dead cells and other debris formed as a result of tissue damage (Neumann et al., 2009). The detrimental effects, on the other hand, have been attributed to the production of pro-inflammatory and cytotoxic mediators (Loane and Kumar, 2016). The temporospatial activation of microglia corresponds with altered morphology, gene expression, and function, which may contribute to the pathophysiology of TBI. Importantly, there is emerging evidence of overlapping phenotypic states that may dictate their functional and morphological response (Dubbelaar et al., 2018) that may vary according to their distribution in the brain and the timing after injury (Loane and Kumar, 2016; Donat et al., 2017; Caplan et al., 2020).

Previous studies have shown that Eph receptor/Ephrin signaling regulates microglia activity in different neuropathological conditions (Deng et al., 2017; Wei et al., 2019). EphB receptor/Ephrin B is the most well-studied signaling in microglia. In murine experimental ocular hypertension, EphB2, EphB3, and Ephrin B3 are upregulated in optic nerve head microglia, and their expression was associated with microglia activation (Du et al., 2007; Fu et al., 2010). In addition, EphrinB2-induced hyperalgesia *via* activation of the spinal cord microglial ERK-5/CREB pathway (Yu et al., 2017). In diabetic neuropathic pain, blocking of spinal EphB1 reduced microglia activation along with inflammatory mediator production and subsequently reduced late diabetes-induced allodynia (Deng et al., 2017). EphA receptor signaling may also contribute to microglia activation and proliferation. In the oxygen-glucose deprivation and reperfusion (OGD/R) model to induce ischemic injury, neuronal EphA4 deficiency inhibits microglia proliferation and promotes their polarization into anti-inflammatory phenotype (Wei et al., 2019). Most of the previous studies used ionized calcium-binding adaptor molecule 1 (Iba1) as a microglia marker. Iba1 is also expressed in peripheral-derived macrophages (PDM), which invade the CNS compartment during inflammation. Hence, discriminating between microglia and PDMs remains an obstacle in determining the role of Eph receptor/Ephrin signaling in resident microglia activation (Du et al., 2007; Ernst et al., 2019).

C-X3-C Motif Chemokine Receptor 1 is a fractalkine receptor expressed in both microglia and hematopoietic cells (Lee et al., 2018). Our previous study showed that the EphA4 receptor is upregulated in C-X3-C Motif Chemokine Receptor 1 (Cx3cr1)-expressing cells in the peri-lesion, following moderate TBI (Kowalski et al., 2019). We also found that the use of bone marrow chimeric EphA4-KO mice ameliorated CCI-induced tissue damage, indicating its possible role in innate immune regulation and TBI pathophysiology (Kowalski et al., 2019). However, its role in regulating the function of resident microglia remains unclear. In the current study, we utilized conditional microglia-specific EphA4 KO mice to study the histopathological outcome and effects on the BBB, following unilateral, moderate CCI injury.

## Methods

### Animals

*Cx3cr1*^*CreER*^ knock-in/knock-out mice that express Cre-ERT2 fusion protein and EYFP were purchased from Jackson Labs (021160; Jackson Laboratory, Bar Harbor, ME). *Cx3cr1*^*CreER*^ mice were backcrossed at least 10 generations using our *EphA4* ^*f*/*f*^ mice [012916; Jackson Laboratory, Bar Harbor, ME (Herrmann et al., 2010)], which we previously backcrossed onto the CD1 background (Okyere et al., 2016, 2020) to produce *Cx3cr1*^*CreER*/+^ *EphA4* ^*f*/*f*^ mice. *Cx3cr1*^*CreER*/*CreER*^*EphA4*^+/+^ (knockout Cx3cr1) and *Cx3cr1*^*CreER*/+^*EphA4*^+/+^ (heterozygous Cx3cr1) mice were also bred on the CD1 background and used as wild-type EphA4 controls. Mice were housed 4–5/cage in an AAALAC-accredited facility with a 12-h (hr) light-dark cycle, and food and water were provided *ad libitum*. The protocol for animal experiments was approved by the Virginia Tech Institutional Animal Care and Use Committee (IACUC; #21-044).

### Genotyping

Genotyping for *Cre, Cx3cr1*, and *EphA4*^*f*/*f*^ detection was performed using tail snips as described previously and recommended by Jackson Labs (Truett et al., 2000). Briefly, 2 mm of the tail was digested in 75-ul 25-mM NaOH in 0.2-mM EDTA at 98°C for 1 h. About 75 ul of 40 mM Tris HCl (pH 5.5) was added, and then, the samples were centrifuged at 5,000 rpm for 2 min. The PCR amplification reaction was performed using the sample supernatant (1 μl), primers described in Table 1 (0.2 μM), and GoTaq Green Master Mix according to the protocol of the manufacturer (Promega, USA).

**Table 1:** Sequence of primers used in genotyping.

**Target gene**	**Primer sequence 5^′^ → 3^′^**
EphA4	F: TGC TAA CAG GCA CTT AGA TCC C
	R: TAA TTG TAA TCA GTG GGC GGG C
Tamoxifen induced	F: GCA CAC TTA GCA ATT CAG TGT GGG
excised EphA4 flox	Mutant R: CCT GCA AAT TAA GGG CAG GAA GAG
	Wild Type R: TAA TTG TAA TCA GTG GGC GGG C
Cre	F: GCG GTC TGG CAG TAA AAA CTA TC
	R: GTG AAA CAG CAT TGC TGT CAC TT
Cx3cr1	F: AAG ACT CAC GTG GAC CTG CT
	Mutant R: CGG TTA TTC AAC TTG CAC CA
	Wild Type R: AGG ATG TTG ACT TCC GAG TTG

### Generation of GFP Bone Marrow Chimeric Mice

Bone marrow chimeric mice were produced as previously described (Holl, 2013; Kowalski et al., 2019). Briefly, male *EphA4*^*f*/*f*^ recipient mice were exposed to two doses of 500 rad spaced 6 h apart using x-ray irradiation (Radsource, Brentwood, TN, USA). At 24-h post-irradiation, the mice received bone marrow cells isolated from age-matched male *Rosa26*^*mtmg*^/*Tie2Cre* donors (Okyere et al., 2016). Each recipient mouse received 100-μl cell suspension with 1–5 million cells *via* tail vein injection. The mice were maintained on gentamycin sulfate water (1 mg/ml) for 3 days before irradiation and 2 weeks after transplantation.

### Controlled Cortical Impact Injury

CCI was performed as previously described (Brickler et al., 2016, 2018; Hazy et al., 2019). The mice were intraperitoneally injected with tamoxifen (100 mg/kg) for 5 consecutive days, and then subjected to CCI injury at 30 days after the last injection or 30 days post-adoptive transfer. Briefly, the mice were anesthetized by subcutaneous injection of ketamine (100 mg/kg) and xylazine (10 mg/kg), and then placed and secured in a stereotaxic frame. Body temperature was maintained at 37°C during the surgery. The scalp was shaved and sanitized, and a midline incision was made to expose the skull. A craniectomy 4-mm diameter was made using a portable drill. Injury was induced using 5. m/s velocity, 2-mm depth, and 150-ms duration. The incision was closed using Vetbond tissue adhesive (3M, Saint Paul, MN). At the end of the experiment, the mice were euthanized by the toxic dose of isoflurane followed by decapitation for microglia isolation or by ketamine/xylene cocktail followed by perfusion fixation.

### Blood Sampling and Microglia Isolation

*Cx3cr1*^*CreER*/+^ *EphA4* ^+/+^ and *Cx3cr1*^*CreER*/+^ *EphA4* ^*f*/*f*^ mice (naïve or 2 hpi) were euthanized with the lethal dose of isoflurane. Blood samples were drawn from the heart in heparinized tubes, and then the mice were perfused with 10-ml PBS to flush out the blood from the circulation. The blood samples were subjected to hypotonic lysis to deplete the red blood cells, washed two times with PBS and then suspended in TRIzol® reagent for RNA extraction. Brains were removed and the cortices were microdissected in HBSS (without Ca or Mg) on ice and dissociated using the neural tissue dissociation kit (Miltenyi Biotec, MA, USA) according to manufacture protocol. After dissociation, cell pellets were suspended in complete DMEM media containing 10% FBS, 100 units/ml penicillin G, and 100-μg/ml streptomycin, and then plated in a non-tissue culture-treated 100-mm plate. Cells were incubated for 45 min at 37°C, 95% humidity, and 5% CO_2_, conditions that allow only microglia to adhere. Non-adherent brain cells were collected, centrifuged at 400 × g for 5 min, washed two times with PBS, and then pellets were suspended in TRIzol® reagent (Ambion) for total RNA or DNA extraction. Adherent microglia were washed two times with PBS and then placed in TRIzol® reagent for total RNA and DNA extraction. PCR amplification was performed using a 1-μl DNA sample, 0.2-μM primers (described in Table 1), and the GoTaq Green Master Mix according to the protocol of the manufacturer (Promega, USA).

### Quantitative Real-Time PCR

Relative mRNA expression was measured using total RNA (1,000 ng), treated with DNAse I (Sigma Aldrich, St. Louis, MO, USA), and then cDNA was synthesized using the iScript™ cDNA synthesis kit (Biorad, Hercules, CA). cDNA (50 ng) was amplified using iTaq™ Universal SYBR® Green Supermix (Biorad, Hercules, CA, USA). Primers used are outlined in Table 2. ΔCT values for target genes were calculated relative to GAPDH internal control, and fold change in mRNA expression was calculated using the 2^**–**ΔΔCT^ method.

**Table 2:** Sequence of primers used in qRT-PCR.

**Target gene**	**Primer sequence 5^′^ → 3^′^**
*GAPDH*	F: CGT CCC GTA GAC AAA ATG GT
	R: TCA ATG AAG GGG TCG TTG AT
*EphA4*	F: AAA AAT GTA CTG TGG GGC AGA T
	R: TCC GTG GAA AGA GCT TTG TAA T
*Cx3cr1*	F: GTG AGT GAC TGG CAC TTC CTG
	R:AAT AAC AGG CCT CAG CAG AAT C
*TMEM119*	F: AGA CCC TTC TGC TTC CCC TT
	R: CCC AGT ATG TGG GGT CAC TG
*NeuN*	F: CAC TCT CTT GTC CGT TTG CTT C
	R: CTG CTG GCT GAG CAT ATC TGT A
*GFAP*	F: ACC AGT AAC ATG CAA GAG ACA GAG
	R: GAT AGT CGT TAG CTT CGT GCT TG
*VE-cadherin*	F: AGG ACA GCA ACT TCA CCC TCA
	R: AAC TGC CCA TAC TTG ACC GTG

### Perfusion Fixation and Brain Serial Sectioning

The mice were injected with 2,000 units/kg heparin and then euthanized using 150-mg/kg ketamine and 15-mg/kg xylazine cocktail. After losing the pedal reflex, cardiac perfusion was performed using Gilson MiniPuls3 peristaltic perfusion pump (Gilson Scientific, Bedfordshire, UK) at a rate of 5 ml/min. To flush the blood from the circulation, 10 ml of heparin (20 unit/ml) diluted with 1× PBS was perfused through the left ventricle after making an incision in the right atrium, followed by perfusion of 50 ml of ice-cold 4% paraformaldehyde (PFA) in PBS, pH 7.5. Brains were cryopreserved using a sucrose gradient, snap-frozen and embedded in Tissue Tek O.C.T. (Sakura, Torrance, CA), and then stored at −80°C until sectioning. Serial coronal sections were collected (−1.1- to −2.6-mm A/P) using a cryostat (CryoStar NX70, Thermo Fisher Scientific, MA, USA). A total of five tissue sections (30 μm) were mounted on charged slides spaced 450 μm apart, heat dried, and then stored at −80°C.

### Lesion Volume and Cortical Mantle Thickness Analysis

Perfused-fixed, serial coronal brain sections were postfixed with 10% formalin (ThermoFisher Scientific, Waltham, MA, USA), washed with 1X PBS, and incubated with 0.2% cresyl violet solution (Electron Microscopy Sciences, Hatfield, PA, USA) for 30 min. Slides were washed with deionized water, dehydrated gradually with serial ethanol concentrations, and then immersed in xylene and mounted with Permount® mounting media (ThermoFisher). Lesion volume was measured blindly as previously described (Kowalski et al., 2019). The slides were imaged using an upright Olympus microscope BX51TRF (Olympus America, Center Valley, PA, USA) at 4× magnification, and the volume of injured tissues (mm^3^) was estimated in the ipsilateral cortex of five serial coronal sections of each brain using Cavalieri Estimator from StereoInvestigator (MicroBrightField, Williston, VT, USA).

### Blood-Brain Barrier Permeability Measurement

Immunoglobulin-G deposition volume was measured in perfused fixed brain sections. Serial coronal sections were blocked and permeabilized with 2% cold water fish skin gelatin (Sigma, Inc., St. Louis, MO) in 0.2% triton-X100 for 1 h and then incubated in donkey anti-mouse IgG 594 (Thermofisher, MA, USA) diluted in blocking solution (1:250). The slides were then washed three times 15 min each with 1X PBS and then mounted with media containing DAPI counterstain (SouthernBiotech, Birmingham, AL). Evans Blue dye extravasation was measured as previously described (Kowalski et al., 2019). Briefly, the mice were injected with 2.% filter-sterilized Evan's blue (EB) solution (4 ml/kg of body weight) *via* tail vein. Three h later, the mice were euthanized using the lethal dose of isoflurane followed by decapitation. Cortices from ipsilateral and contralateral hemispheres were dissected and incubated in 500-μl Formamide (Invitrogen, 15515-026) for 24 h at 55°C with continuous shaking. Samples were centrifuged at 12,000×*g* for 10 min, and absorbance was measured in the supernatant at 610 nm using a plate reader (Synergy HTX, multi-mode reader, BoiTek).

### Immunohistochemical Analysis

Coronal sections were air-dried, washed with 1X PBS, blocked with 2% cold water fish skin gelatin (Sigma, Inc., St. Louis, MO, USA) in 0.2% triton-X100 for 1 h, incubated with a primary antibody diluted in blocking solution overnight at room temperature (RT), and then washed with 1X PBS six times 10 min each and incubated with appropriate secondary antibody solution (1:250 dilution) for 1 h at RT. Slides were then washed with 1X PBS and then mounted with media-containing DAPI counterstain (SouthernBiotech, Birmingham, AL, USA). Antibodies used are goat anti-EphA4 at 1:100 dilution (R&D systems, Minneapolis, MN, USA), rabbit anti-TMEM119 at 1:100 dilution (Abcam, Waltham, MA, USA), rabbit anti-CCR2 at 1:100 dilution (Abcam, Waltham, MA, USA), rat anti-CD68 at 1:100 dilution (Thermofisher, USA), rabbit anti-Iba1 at 1:200 dilution (FUJIFILM Wako Chemicals U.S.A. Corporation), donkey anti-goat 594 (Thermofisher, USA), donkey anti-rabbit 647 (Thermofisher, USA), donkey anti-rabbit 555 (Thermofisher, USA), and donkey anti-rat 647 (Thermofisher, USA). Image acquisition was performed using a Zeiss 880 confocal microscope (Carl-Zeiss, Oberkochen, Germany).

### Statistical Analysis

Data are presented as mean ± standard error of mean (SEM). Statistical analysis was performed using the GraphPad software (La Jolla, CA, USA). The intergroup variations were analyzed using Student's unpaired two-tailed *t*-test (to compare between two experimental groups) and one-way ANOVA, followed by Bonferroni's test (to compare multiple groups). The variations were considered significant at *p* < 0.05.

## Results

### CCI Increased Ipsilateral EphA4 Expression in CX3CR1-Expressing Cells

**Figure 1:**
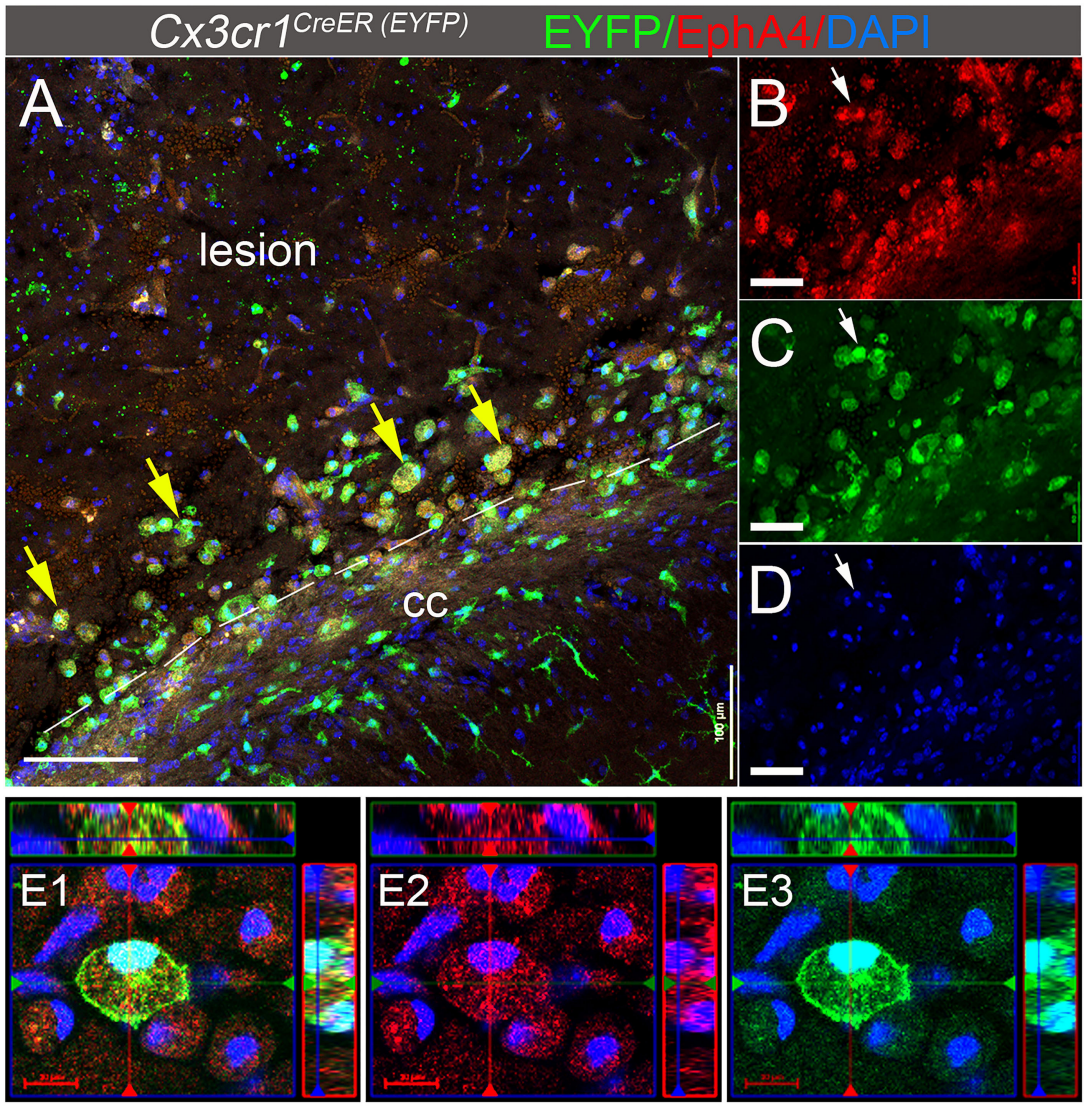
EphA4 is upregulated on Cx3cr1-expressing cells in the peri-lesion 3 days post CCI injury. **(A–D)** Representative confocal images at max z-projection for immunohistochemical analysis for EphA4 (red), EYFP in Cx3cr1-expressing cells (green), and DAPI (blue) at 3 dpi in the ipsilateral cortex of *Cx3cr1*^*CreER*/+^mice. CCI injury increased EphA4 expression in cx3cr1^+^ cells (arrows) in the peri-lesion areas. **(E1–E3)** Co-localization of EphA4 and EYFP in amoeboid-shaped microglia and/or PDM. Scale bar: **(A)** 100 μm; **(B–D)** 50 μm and **(E1–E3)** 20 μm.

We have previously reported that Cx3cr1-expressing cells in the peri-lesion show high levels of EphA4 1 day-post CCI injury (dpi) in *Cx3cr1*^*GFP*/+^ knock-in mice (Kowalski et al., 2019). In the current study, we used *Cx3cr1*^*CreER*^ mice, which constitutively express EYFP in Cx3cr1-positive microglia and peripheral-derived immune cells. Consistent with our previous findings, induction of CCI injury in the EphA4 wild type (WT) *Cx3cr1*^*CreER*/+^ *EphA4*^+/+^ mice increased EphA4 expression on Cx3cr1^EYFP+^ cells located in the peri-lesion at 3 dpi. Immunohistochemical analysis showed the co-localization of EYFP (Cx3cr1^+^, green fluorescence) and EphA4 (red fluorescence) in the peri-lesion, indicating that CCI injury increased EphA4 expression in microglia and/or peripheral-derived immune cells (Figures 1A–E). Interestingly, EphA4 was expressed on amoeboid Cx3cr1-expressing cells but not in ramified-resting microglia (Supplementary Figure 1), indicating the possible role of EphA4 in microglia activation and acute tissue damage after acute CCI injury.

### Microglial EphA4 Expression in the Damaged Cortex After CCI Injury

**Figure 2:**
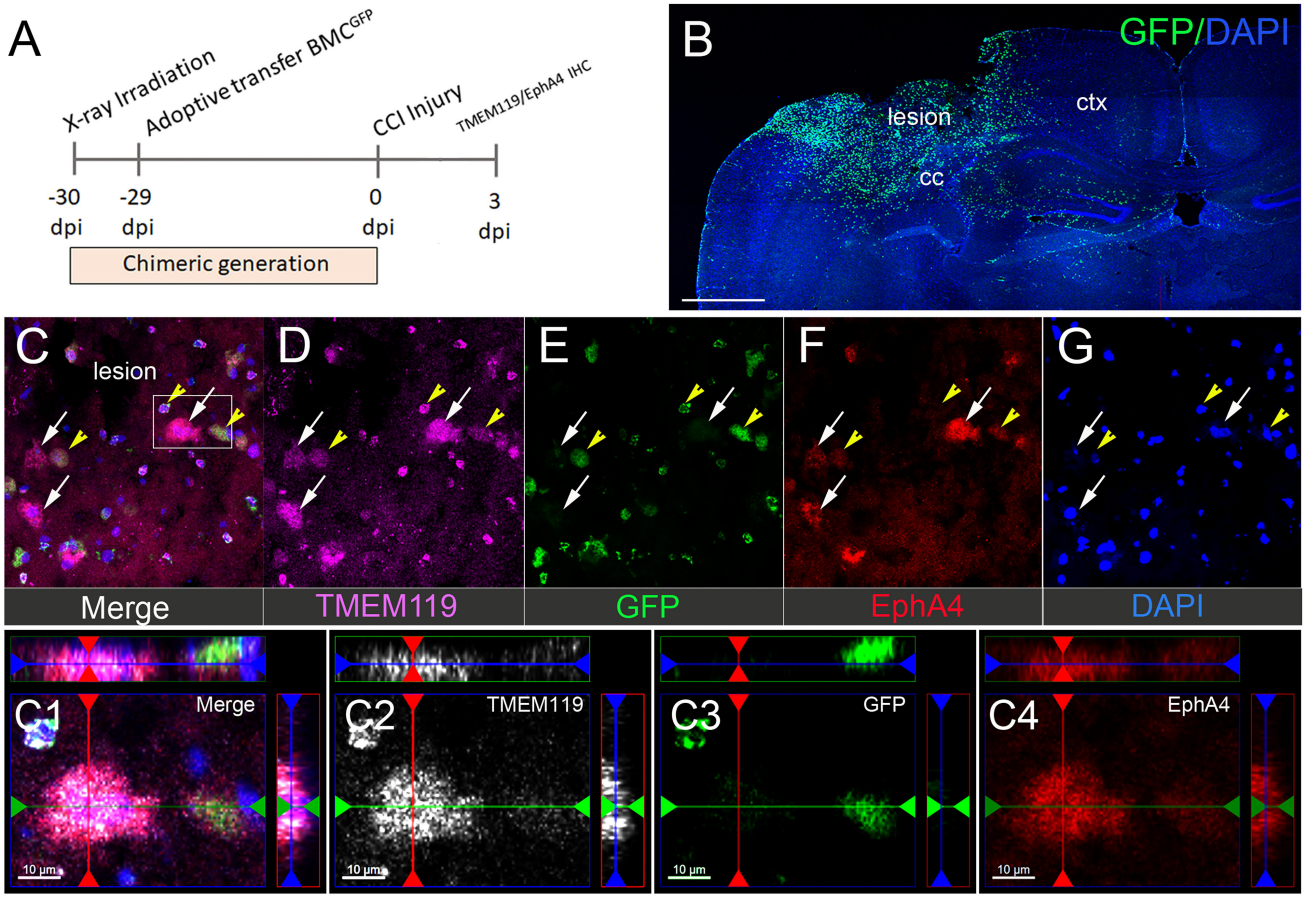
Microglial EphA4 is upregulated in the damaged cortex after CCI injury. **(A)** Timeline for generation of GFP bone marrow chimeric mice and CCI injury. Mice were euthanized 3 dpi, and brain sections were used for IHC analysis of TMEM119 and EphA4. **(B)** Representative confocal images for peripheral-derived immune cells (GFP), infiltrating the ipsilateral injured cortex. **(C–G)** Representative images of TMEM119 (purple) and EphA4 (red). EphA4 is expressed on amoeboid-resident microglia (white arrow, EphA4^+^/GFP^−^/TMEM119^+^) as well as peripheral-derived GFP immune cells, which also express TMEM119 (yellow arrowhead, EphA4^+^/GFP^+^/TMEM119^+^). **(C1–C4)** Representative images showing the colocalization of TMEM119 (white) and EphA4 (red) in GFP^−^ microglia. Scale bar in **(B)** 1,000 μm; **(C1–C4)** 10 μm.

To distinguish between resident microglia and peripheral-derived macrophages, we used GFP bone marrow-chimeric mice (Kowalski et al., 2019). To label the microglia, we used antibodies against TMEM119, a well-established microglia marker (Satoh et al., 2016; Li et al., 2018). At 3 dpi, we observed a substantial influx of peripheral-derived GFP^+^ immune cells into the lesion core and the peri-lesion cortex (Figures 2A,B) that was not present in the contralateral hemisphere (Supplementary Figure 2). The GFP^+^ immune and TMEM119^+^ (Figures 2C,D; white arrows) cells displayed EphA4 expression in the cortical lesion (Figure 2C). Surprisingly, we found numerous GFP^+^/TMEM119^+^ co-labeled cells (Figures 2C–G; yellow arrows), indicating that TMEM119 transmembrane protein expression is induced on recruited GFP+ peripheral-derived immune cells in the damaged cortex and is, therefore, not specific to microglia (Satoh et al., 2016; Li et al., 2018) in the murine brain under the condition of CCI injury. Consistent with our finding in Figure 1, we also observed that EphA4 is upregulated on amoeboid microglia (TMEM119^+^/GFP^−^) in the lesion core and at the boundary of the peri-lesional cortex after acute injury (Figures 2C1–C4).

### Generation of Microglia-Specific EphA4-Deficient Mice

**Figure 3:**
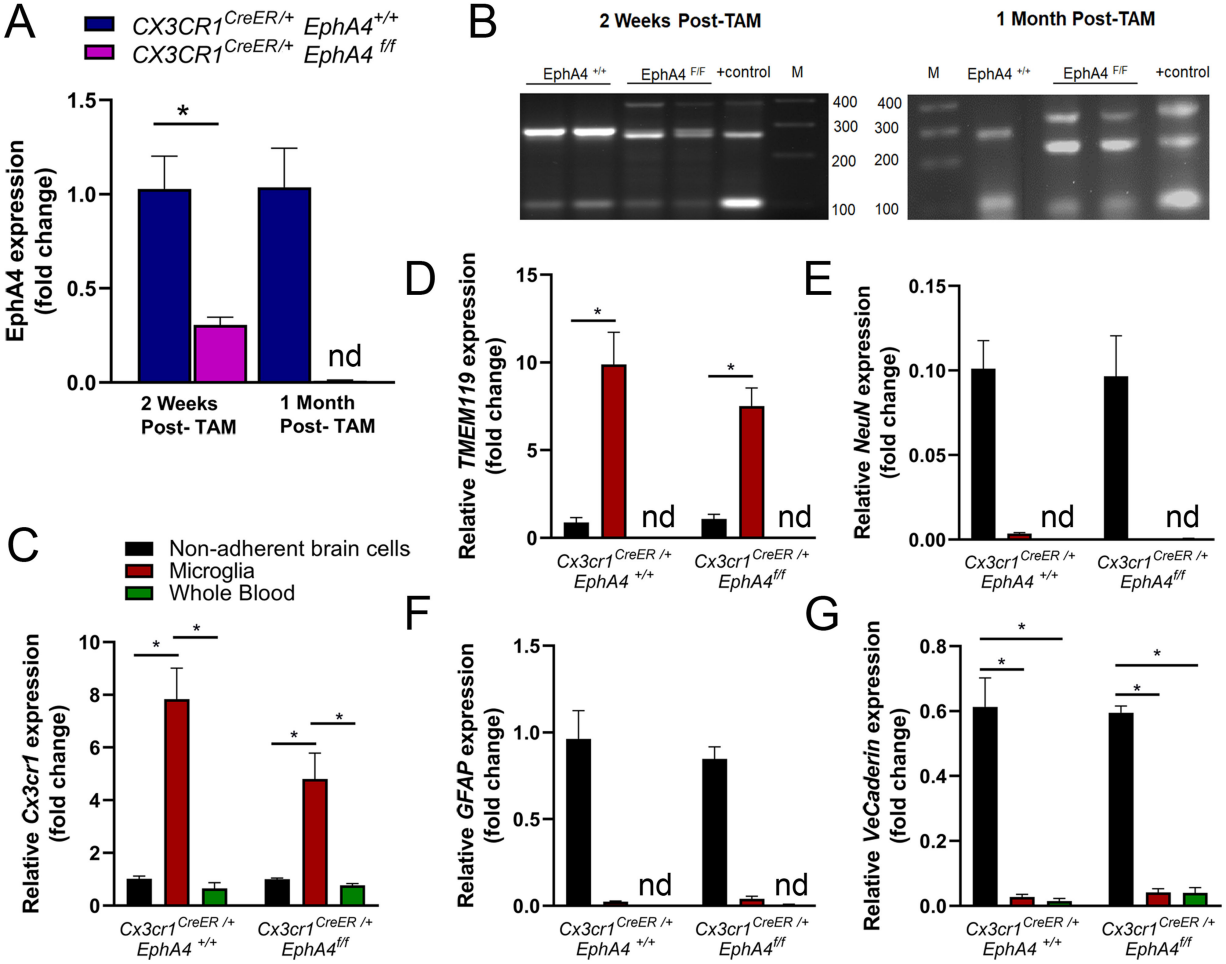
Generation of microglia-specific EphA4-deficient mice. **(A)** Relative EphA4 expression in cortical microglia of naive *Cx3cr1*^*CreER*/+^*EphA4* ^+/+^ and *Cx3cr1*^*CreER*/+^*EphA4* ^*f*/*f*^ was measured at 2 weeks and 1-month post tamoxifen (TAM) injection using qRT-PCR. **(B)** PCR amplification of EphA4^−^WT allele (286 bp), loxP-flanked EphA4 allele (390 bp), EphA4-excised allele (250 bp), and Cre (100 bp) in the DNA extracted from *Cx3cr1*^*CreER*/+^*EphA4*^+/+^ and *Cx3cr1*^*CreER*/+^*EphA4*^*f*/*f*^ microglia at 2 weeks and at 1-month post-tamoxifen injection. Positive (+) control showing loxP-flanked EphA4 allele (390 bp), EphA4-excised allele (250 bp), and Cre (100 bp). *M* = 100 bp low-scale DNA ladder. (C-G**)**qRT-PCR analysis for microglial genes (*Cx3cr1*, **C** and *TMEM119*, **D**), neuronal gene (*NeuN*, **E**), astrocyte gene (*GFAP*, **F**), and vascular endothelial gene (*VE-cadherin*, **G**) in isolated microglia, non-adherent remaining cortical cells, as well as whole blood collected from naïve *Cx3cr1*^*CreER*/+^*EphA4*^+/+^ and *Cx3cr1*^*CreER*/+^*EphA4*^*f*/*f*^ mice. *N* = 3–6, ^*^p < 0.05, significant difference between the designated groups; nd, not detected.

To determine the role of EphA4 in CCI-induced microglia activation and acute tissue damage, we sought to generate microglia-specific EphA4 deficient mice using *Cx3cr1*^*CreER*^ knock-in/knock-out mice, which express Cre-ER fusion protein and EYFP in microglia and other peripheral immune cells, including monocytes, and then crossed with *EphA4*^*f*/*f*^ *mice to generate Cx3cr1*^*CreER*/+^ *EphA4*^*f*/*f*^ as well as *Cx3cr1*^*CreER*/+^ *EphA4*^+/+^ mice. Five daily consecutive tamoxifen (TAM) injections induced Cre recombinase-mediated deletion of the floxed EphA4 sequence. As a result, relative mRNA expression of EphA4 was reduced in microglia isolated from the cortex at 2 weeks and at 1 month after the last tamoxifen dose (Figure 3A). The activity of Cre recombinase has been confirmed by PCR amplification. *Cx3cr1*^*CreER*/+^ *EphA4*^+/+^ microglia showed two bands (286 bp for EphA4-WTallele and 100 bp for Cre); while *Cx3cr1*^*CreER*/+^*EphA4*^*f*/*f*^ microglia showed three bands (390 for loxP-flanked EphA4 allele, 250 for EphA4-excised allele, and 100 bp for Cre) at 2 weeks and at 1-month post-TAM injection (Figure 3B).

To test the purity of cortical microglia isolates, we measured the mRNA expression of microglial genes (*Cx3cr1* and *TMEM119*), neuronal gene (*NeuN*), astrocyte gene (*GFAP*), and vascular endothelial gene (*VE-cadherin*) in isolated microglia, non-adherent cortical cells, as well as blood immune cells collected from naive WT-*Cx3cr1*^*CreER*−/+^*EphA4*^+/+^ and KO-*Cx3cr1*^*CreER*−/+^*EphA4*^*f*/*f*^ mice at 1-month post-TAM. Relative *Cx3cr1* and *TMEM119* expression were significantly high in microglia when compared to cortical cells and whole blood immune cells (Figures 3C,D, respectively). The expression of *NeuN, GFAP*, and *VE-Cadherin* was negligible or not detected(nd) in both microglia and whole blood immune cells (Figures 3E–G). These data confirm that a highly pure microglia population was isolated and used for EphA4 detection.

Cx3cr1 is also expressed in bone marrow-derived immune cells, including monocytes (Lee et al., 2018). Therefore, it is possible that, in addition to deletion in microglia, EphA4 may be deleted from peripheral immune cells in *Cx3cr1*^*CreER*/+^*EphA4*^*f*/*f*^ mice after tamoxifen induction of Cre recombinase. Given the turnover of bone marrow cells, (Yona et al., 2013; Morganti et al., 2019), we measured EphA4 expression in the whole blood at 2 weeks and 1-month post-TAM injection to determine the extent of the conditional deletion of EphA4. Interestingly, we found a trend toward reduced EphA4 expression at 2 weeks and no significant change in expression at 1-month post-TAM injection (Supplementary Figure 3A). Taken together, our data suggest EphA4 is deleted only in microglia of *Cx3cr1*^*CreER*/+^*EphA4*^*f*/*f*^ mice, following 1-month post-tamoxifen regimen.

### Increased EphA4 Expression in Cortical Microglia at 2 h Post-CCI Injury

**Figure 4:**
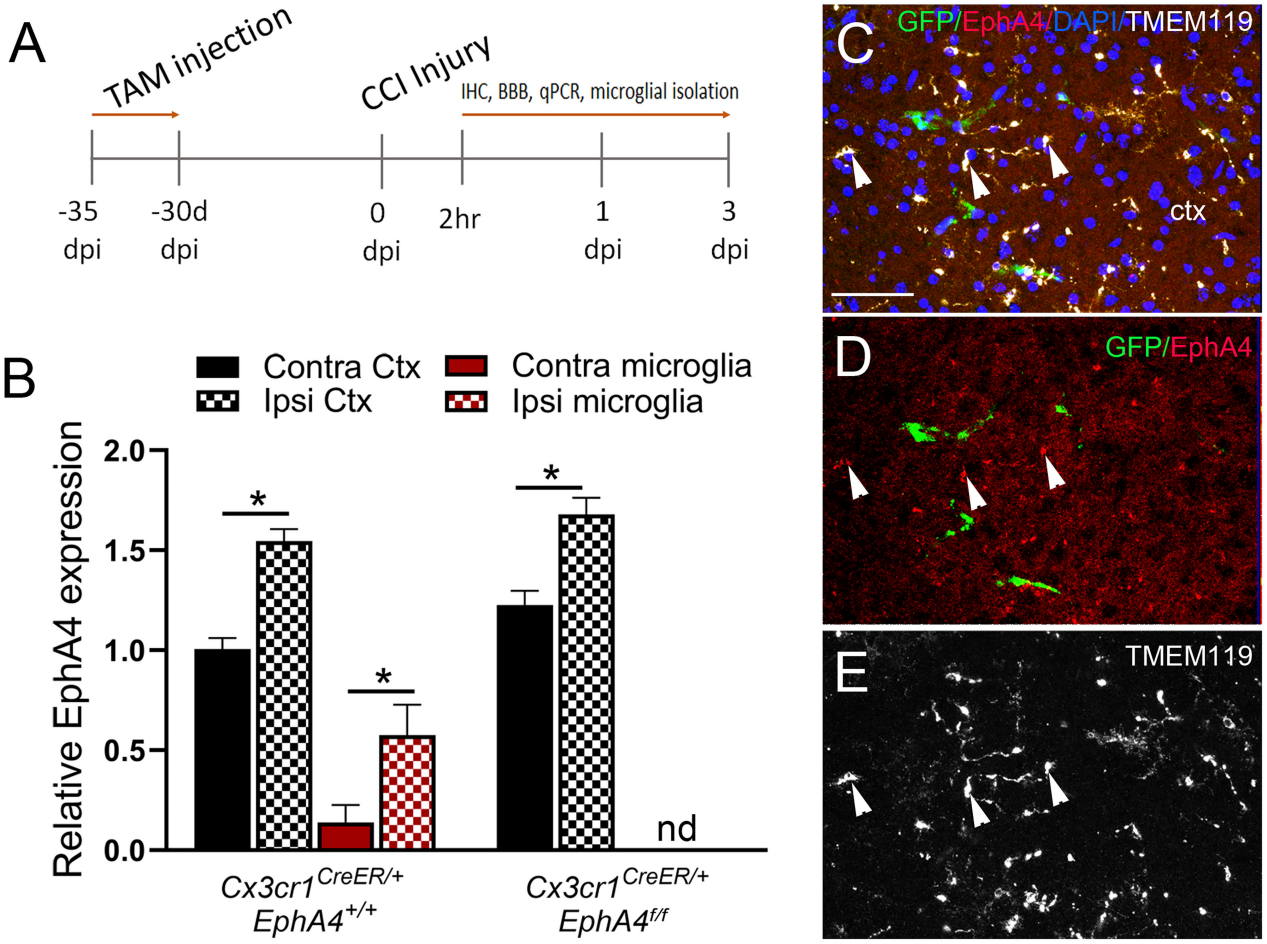
EphA4 expression in microglia at 2 h post-CCI injury. **(A)** *Cx3cr1*^*CreER*/*creER*^*EphA4* ^+/+^, *Cx3cr1*^*CreER*/+^*EphA4* ^+/+^, and *Cx3cr1*^*CreER*/+^*EphA4* ^*f*/*f*^ mice were intraperitoneally injected with five daily doses of tamoxifen (100 mg/kg) and then subjected to CCI injury 30 days after the last injection. Microglia were isolated from ipsilateral and contralateral cortices for qRT-PCR analysis at 2 h. **(B)** Relative EphA4 mRNA expression was measured in isolated microglia and non-adherent cells from ipsilateral (ipsi) and contralateral (contra) cortices (Ctx) at 2 h. *N* = 5, ^*^p < 0.05, significant difference between the designated groups; nd, not detected. **(C–E)** Representative confocal images for IHC analysis of TMEM119 (white) and EphA4 (red) in GFP bone marrow chimeric WT mice, showing increased EphA4 expression in resident microglia (white arrowhead, EphA4^+^GFP^−^TMEM119^+^). Scale bar = 100 μm.

To determine if conditional microglia-specific EphA4 deficiency influences microglia activation and improves the acute histopathological outcomes of CCI injury, the mice were injected with TAM (five daily doses) and then subjected to CCI injury 1 month after the last tamoxifen dose (Figure 4A). About 2 h post-CCI injury (2 h), microglia were isolated from the ipsilateral and contralateral cortices, and then EphA4 transcript was quantified using qPCR. We found EphA4 was upregulated in the ipsilateral microglia and microglia-depleted cortical cells of *Cx3cr1*^*CreER*/+^*EphA4*^+/+^ mice when compared to contralateral. In *Cx3cr1*^*CreER*/+^*EphA4*^*f*/*f*^ mice, EphA4 was also upregulated in ipsilateral microglia-depleted cortical cells when compared to contralateral and was not detected in microglia, further confirming genetic microglial-specific knockout (Figure 4B). To confirm changes in protein expression at 2 h, specifically on microglia, we analyzed EphA4 using WT GFP chimeric mice and performed confocal imaging of GFP (green), EphA4 (red), and TMEM119 (white) (Figures 4C–E; white arrowheads). Consistent with qPCR findings, IHC showed that EphA4 was upregulated on microglia (GFP^−^/TMEM119^+^) in the injured cortex. These findings indicate EphA4 is present on microglia as early as 2 h post-CCI injury.

### Deficiency in Microglial EphA4 Has No Effect on Acute Tissue Damage and BBB Disruption Following CCI Injury

**Figure 5:**
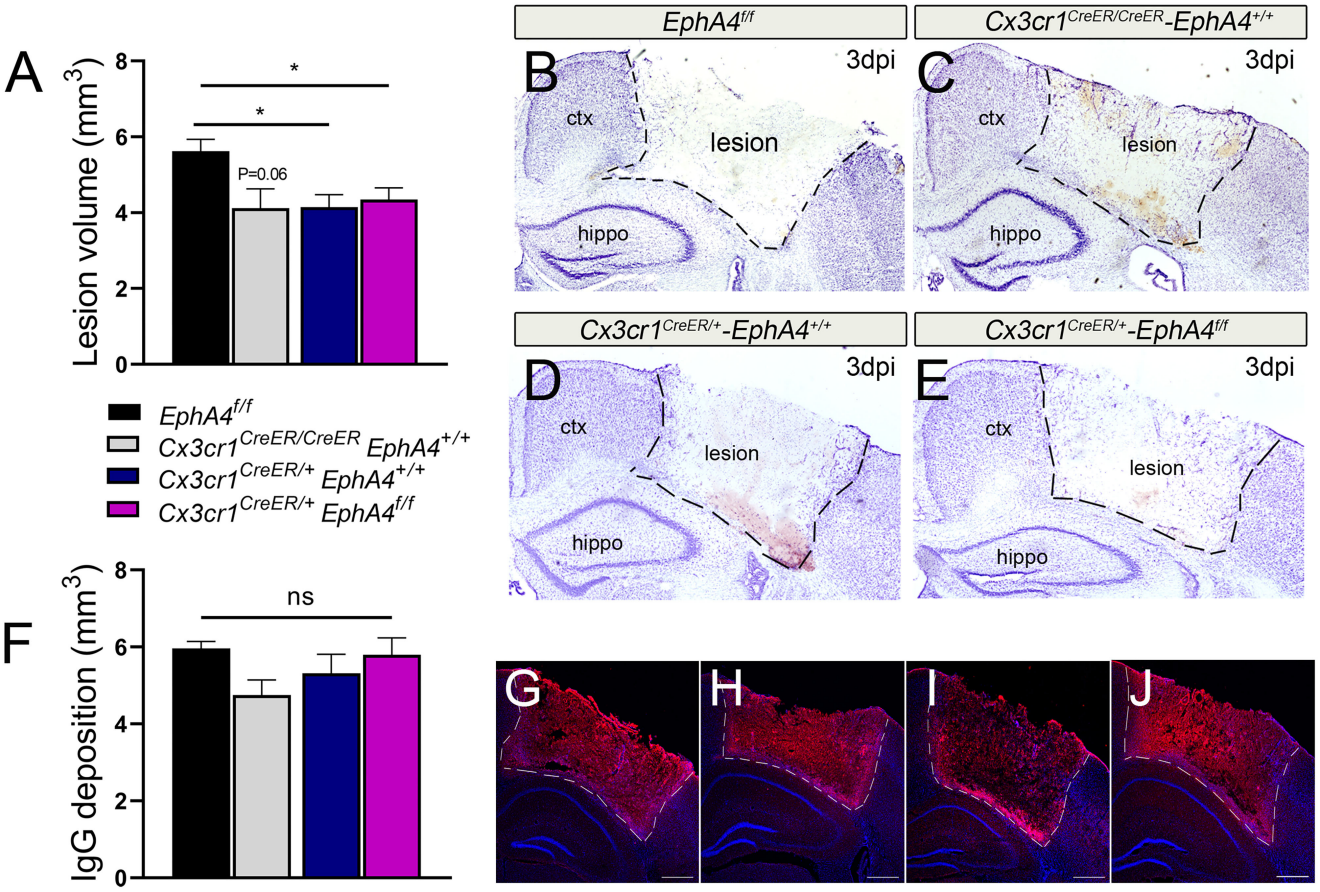
Conditional deletion of microglial *EphA4* does not affect acute tissue damage or BBB disruption, following CCI injury. **(A)** Lesion volume (mm^3^) 3 dpi was estimated in the ipsilateral cortex of five Nisslstained serial coronal sections of each brain using Cavalieri Estimator from StereoInvestigator. **(B–E)** Representative mosaic images for the ipsilateral cortices of Nissl-stained sections taken at 4× magnification. **(F)** Immunoglobulin G (IgG) deposition volume was measured at 3 dpi in the ipsilateral cortex of five serial coronal sections of each brain using Cavalieri Estimator from StereoInvestigator. **(G–J)** Representative confocal images for the ipsilateral cortices of anti-mouse IgG-stained sections (red) taken at 4× magnification. *N* = 5–15, ^*^p < 0.05, significant difference between the designated groups. ns, nonsignificant difference was observed.

To examine the effects of microglia-specific EphA4 knockout on the TBI outcome, we quantified the lesion volume and BBB permeability at 3 days post-CCI injury (dpi). The insertion of the Cre-ERT2 and EYFP to the endogenous *Cx3cr1* promoter/enhancer element in *Cx3cr1*^*CreER*^ knock-in/knock-out mice reduces endogenous CX3CR1 expression. Hence, we chose to use heterozygous *Cx3cr1*^*CreER*/+^*EphA4*^+/+^, homozygous *Cx3cr1*^*CreER*/*CreER*^*EphA4*^+/+^ (Supplementary Figure 3B), and *EphA4*^*f*/*f*^ as EphA4 wild-type control mice to compare to *Cx3cr1*^*CreER*/+^*EphA4*^*f*/*f*^ microglial EphA4-KO mice to establish any contribution that Cx3cr1 may also play in CCI-induced brain injury. No significant difference in body weight or cortical mantle thickness exists between these groups of mice (Supplementary Figures 3C–H). Lesion volume was performed on Nissl-stained serial sections using the Cavalieri probe in StereoInvestigator software (Kowalski et al., 2019). Interestingly, no significant difference was observed in the lesion volume of *Cx3cr1*^*CreER*/+^*EphA4*^*f*/*f*^ when compared to either *Cx3cr1*^*CreER*/+^*EphA4*^+/+^ or *Cx3cr1*^*CreER*/*CreER*^*EphA4*^+/+^ (Figures 5A–E). However, a significant difference was found between *EphA4*^*f*/*f*^ and *Cx3cr1*^*CreER*^ mice, suggesting that fractalkine receptor deficiency is acutely neuroprotective, as previously reported (Zanier et al., 2016). Next, we measured BBB permeability using IgG deposition. Slight reduction in IgG deposition volume was observed in *Cx3cr1*^*CreER*/*CreER*^*EphA4*^+/+^ when compared with *EphA4* ^*f*/*f*^; however, no significant difference was observed in *Cx3cr1*^*CreER*/+^*EphA4*^*f*/*f*^ when compared with either *Cx3cr1*^*CreER*/+^*EphA4*^+/+^ or *Cx3cr1*^*CreER*/*CreER*^*EphA4*^+/+^ (Figures 5F–J). We further measured Evans blue extravasation, as previously described (Brickler et al., 2018). Our findings show a significant increase in BBB permeability in the ipsilateral cortex when compared to the contralateral at 3 dpi across all groups of mice (Figure 5F). No significant difference was observed between the groups (Supplementary Figure 4). These data indicate that CCI-induced tissue damage and BBB disruption are not influenced by EphA4 deficiency in resident microglia.

### Deficiency in Microglial EphA4 Does Not Influence Microglia Morphology Following CCI Injury

**Figure 6:**
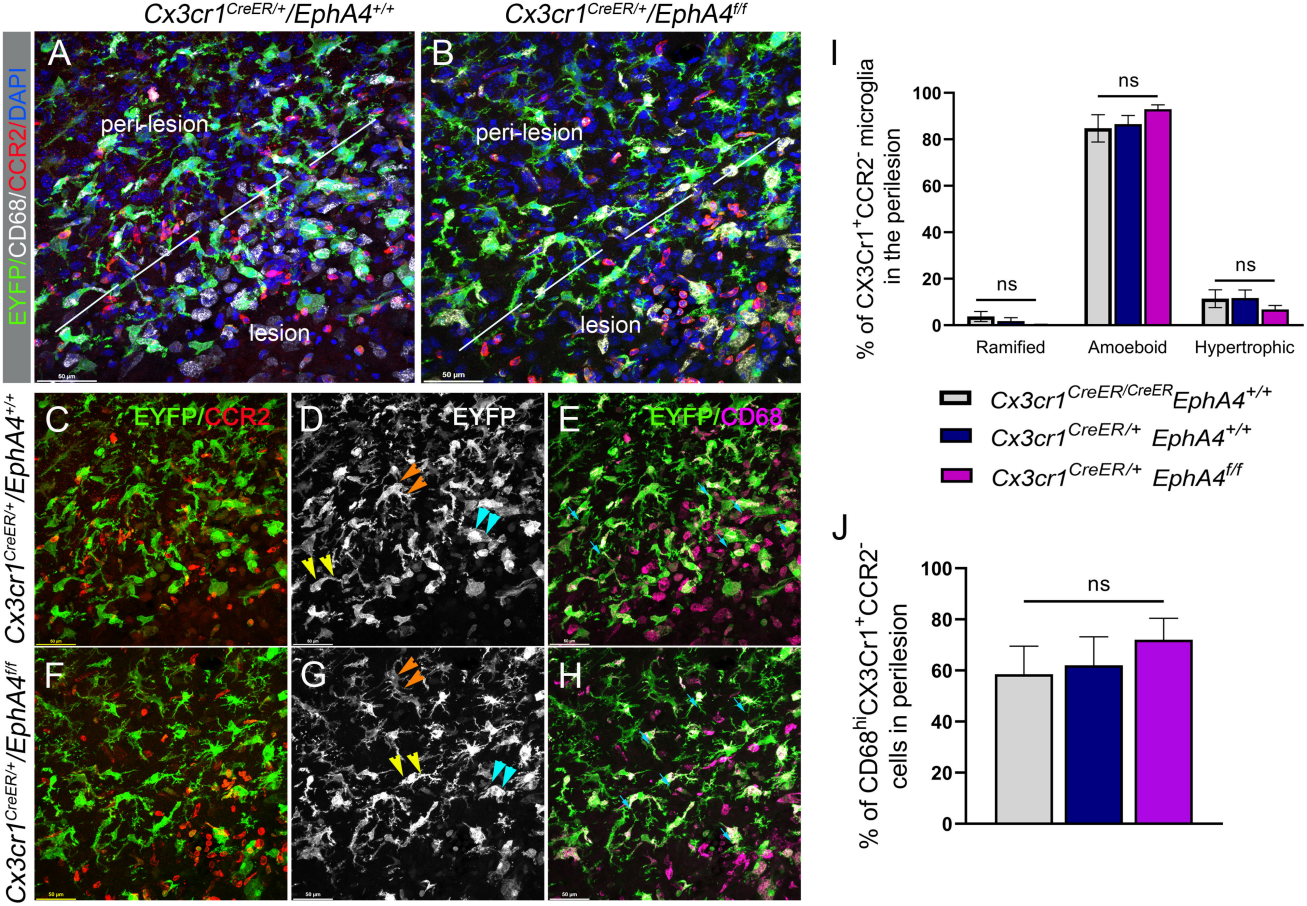
Conditional deletion of microglial *EphA4* does not influence microglia activation or ramification, following CCI injury. **(A, B)** Representative confocal images at max z-projection for immunostaining of CCR2 (red) and CD68 (white) in the cortical lesion and peri-lesion of *Cx3cr1*^*CreER*/+^*EphA4* ^+/+^ and *Cx3cr1*^*CreER*/+^*EphA4* ^*f*/*f*^ mice at 3 dpi. **(C, F)** Co-localization of CCR2 (red) with Cx3cr1-EYFP (green), showing EYFP^+^ CCR2^−^ resident microglia in the peri-lesion. **(D, G)** Microglia ramification in the peri-lesion. EYFP^+^ CCR2^−^ microglia are either ramified (orange arrowhead), hypertrophic (yellow arrowhead), or amoeboid (cyan arrowhead). **(E, H)** Representative confocal images, showing CD68 expression (purple) in activated microglia (CD68^+^/Cx3cr1^+^/CCR2^−^, cyan arrow). **(I)** Quantification of the percentage of resting ramified, activated hypertrophic and activated amoeboid microglia (Cx3cr1^+^/CCR2^−^) in the peri-lesion. **(J)** Quantification of the percentage of CD68^hi^ microglia (CD68^hi^/Cx3cr1^+^/CCR2^−^) in the peri-lesion. *N* = 4–5; ns, nonsignificant difference was observed between different groups.

Upregulation of EphA4 on amoeboid, but not resting ramified, shaped CX3CR1-positive cells (see Figure 1), led us to evaluate whether EphA4 may regulate microglial morphology in response to CCI injury. To test this, we quantified the number of amoeboid, hypertrophic, ramified, and CD68^hi^ (a lysosomal marker associated with phagocytic activity) microglia in the peri-lesion of EphA4 wild type (*Cx3cr1*^*CreER*/+^*EphA4*^+/+^ and *Cx3cr1*^*CreER*/*CreER*^*EphA4*^+/+^) and microglial-specific EphA4-KO mice (*Cx3cr1*^*CreER*/+^*EphA4*^*f*/*f*^) at 3 dpi (Figure 6). Serial coronal sections were used to analyze EYFP^+^Cx3Cr1^+^ cells, following immunostaining with anti-CD68 and anti-CCR2 antibodies. CD68 is a lysosomal protein, which is overexpressed in both macrophages and activated microglia. CCR2 is expressed exclusively on peripheral-derived monocytes/macrophages but not resident microglia (Li et al., 2018) and was, therefore, used in our experiment to distinguish between the two populations. In this case, EYFP+ (Cx3cr1-expressing cells; green) that co-labeled with CCR2 (red) is considered peripheral derived, while EYFP^+^/CCR2^−^ were classified as microglia (Figures 6A,B). Analysis of EYFP^+^/CCR2^−^microglia morphology showed ~99% of cells were ramified in the contralateral cortex (data not shown). However, this was reduced in the ipsilateral (~5%) peri-lesion, while amoeboid and hypertrophic states predominated. No significant difference in the percentage of amoeboid, hypertrophic, or ramified morphology was found between the groups (Figure 6I). We further quantified the percentage of EYFP^+^/CCR2^−^microglia that were CD68^hi^. Our data showed no significant difference in the percentage of CD68-positive microglia (CD68^hi^EYFP^+^ CCR2^−^) between the experimental groups (Figure 6J). These data suggest that EphA4 is indispensable in the regulation of microglia morphology and CD68 expression, following acute CCI injury.

## Discussion

Traumatic brain injury is a major cause of disabilities and deaths from acute injuries in the US and worldwide. The secondary brain injury produced as a result of neuroinflammation determines the severity of TBI outcomes (Flanagan, 2015). As a first line player in the innate immune response, activated microglia contribute to neuroinflammation in TBI (Loane and Kumar, 2016). Previous findings in our lab showed that induction of TBI using the CCI injury model upregulated EphA4 in Cx3cr1-expressing cells in the peri-lesion without discriminating between resident microglia and peripheral-derived immune cells (Kowalski et al., 2019). In the current study, we used GFP bone marrow-chimeric mice to distinguish between Iba-1-expressing peripheral-derived macrophages (PDMs) vs. resident microglia and found that CCI injury increased EphA4 expression in resident microglia as early as 2 h post-injury. To study the role of microglial EphA4 in CCI injury, we used microglia-specific EphA4 KO mice, *Cx3cr1*^*CreER*/+^*EphA4*^*f*/*f*^ and found that conditional deletion did not improve CCI-induced acute tissue damage or BBB disruption. Despite the negative results, our findings help improve our mechanistic understanding of the key players that regulate innate immune involvement in neuroinflammation as a result of TBI. These findings also prevent replication of work and further emphasize the importance of reporting negative results (Mehta, 2019).

*Cx3cr1*^*GFP*/+^ knock-in and *Cx3cr1*^*CreER*/+^*EphA4*^+/+^ mice both show increased EphA4 expression in the perilesional amoeboid shaped Cx3cr1+ cells, which represent either PDMs or activated resident microglia. Given that the two cell populations have distinct functions, we sought to distinguish between microglia and PDMs. Previous studies have used Iba-1 to study the role of Eph receptor/Ephrin signaling in microglia; however, this molecule is also expressed on PDMs, which invade the CNS compartment during inflammation and cannot be used to differentiate between the two cell types (Du et al., 2007; Ernst et al., 2019). The precise distinction between resident microglia and infiltrating myeloid cells is difficult because they share several markers, including Cx3cr1, Iba-1, CD45, and CD11b (Jurga et al., 2020). Although previous transcriptomic and genomic analysis revealed that some signature genes can be used to identify microglia, the expression levels of these genes change according to their function in different pathological conditions (Wes et al., 2016). The two most commonly used microglia markers are purinergic receptor (P2RY12) and transmembrane protein 119 (TMEM119) (Satoh et al., 2016; Honarpisheh et al., 2020). Following TBI, P2RY12 is downregulated on microglia in the injured hemisphere and may not be a suitable microglia marker, especially after brain injury (Toledano Furman et al., 2020). Therefore, in the present study, we used GFP+ bone marrow chimeric mice along with immunostaining for TMEM119 to discriminate between infiltrating PDMs and activated resident microglia, following CCI injury. We found that EphA4 expression is increased in the resident-activated microglia (GFP^−^/TMEM119^+^) in the ipsilateral cortex at 3 dpi. Surprisingly, some of the infiltrating GFP+ cell in the core of the injury express TMEM119. Although further studies are still required to identify the type of bone marrow-derived cells-expressing TMEM119, our observation provides a potential caveat of using TMEM119 as a microglia-specific marker in the injured brain without pre-labeling the infiltrating immune cells.

Previous studies in our lab showed that global deficiency or peptide inhibition of EphA4 reduced tissue damage and inflammation, indicating the important role of EphA4 in the secondary damage induced by TBI (Kowalski et al., 2019). The present study suggests that EphA4 is upregulated in both PDMs and activated microglia, following moderate CCI injury. Unexpectedly, microglial EphA4 deficiency did not improve CCI-induced acute tissue damage, BBB disruption, or alter perilesional microglia activation. Our data verified that Cx3cr1-expressing microglia not peripheral-derived immune cells or other cortical cells are EphA4-deficient in *Cx3cr1*^*CreER*/+^*EphA4*^*f*/*f*^ mice. We also found that CCI injury upregulates EphA4 in cortical cells of *Cx3cr1*^*CreER*/+^*EphA4*^*f*/*f*^ mice; hence, we speculate that EphA4 functions on other cell types such as PDMs might have a greater contribution to the acute pathophysiology of TBI. This postulation is strongly supported by our previous findings, showing that bone marrow chimeric EphA4 KO mice are highly protected against CCI-induced acute tissue injury (Kowalski et al., 2019). However, additional studies are required to investigate the specific role of EphA4 in PDM-mediated effects, following TBI.

Lastly, we cannot rule out the possibility that EphA4 may play a distinct role in the chronic regulation of microglia, which may influence their long-term effects on plasticity, synaptic remodeling, or neuroinflammation. Overall, our findings demonstrate a nonessential role for microglia-specific EphA4 in the acute pathogenesis of TBI.

## Data Availability Statement

The original contributions presented in the study are included in the article/Supplementary Material, further inquiries can be directed to the corresponding author.

## Ethics Statement

The animal study was reviewed and approved by Virginia Tech IACUC; approval #21-044.

## Author Contributions

EB, JM, ES, CK, JJ, AK, NG, EK, ME, MC, and XW performed research and analyzed data. ES and MT wrote and edited the paper, designed research, and contributed reagent/analytic tools. All authors contributed to the article and approved the submitted version.

## Funding

We recognize The Center for Engineered Health for grant support cytometry support. This study was supported by the National Institute of Neurological Disorders and Stroke of the National Institutes of Health, NS096281, NS119540, and NS121103 (MHT).

## Conflict of Interest

The authors declare that the research was conducted in the absence of any commercial or financial relationships that could be construed as a potential conflict of interest.

## Publisher's Note

All claims expressed in this article are solely those of the authors and do not necessarily represent those of their affiliated organizations, or those of the publisher, the editors and the reviewers. Any product that may be evaluated in this article, or claim that may be made by its manufacturer, is not guaranteed or endorsed by the publisher.

## Supplementary Material

Supplementary Figure 1 EphA4 expression on amoeboid not ramified Cx3cr1^+^ cells microglia in the peri-lesion cortex. **(A–D)** Representative confocal images for immunohistochemical analysis for EphA4 (red), EYFP in Cx3cr1-expressing cells (green), and DAPI (blue) at 3 dpi in the ipsilateral cortex of *Cx3cr1*^*CreER*/+^mice. CCI injury increased EphA4 expression in amoeboid cx3cr1+ cells (white arrows) in the lesion and peri-lesion. EphA4 is not observed on ramified microglia (yellow arrowhead). Scale bar = 100 μm.

Supplementary Figure 2 Confocal image analysis of the contralateral cortical hemisphere at 3 dpi in GFP-adoptive transfer mice. **(A)** Merged confocal image max z-projection of Iba1 and GFP in chimeric mice at 3 days post-CCI injury. Individual channel images of GFP **(B)** and Iba1 **(C)**. Scale bar 500 μm. ctx-cortex; cc, corpus callosum.

Supplementary Figure 3 Whole blood EphA4 expression and *Cx3cr1*^*CreER*^ mice genotype, body weight, and cortical mantle thickness. **(A)** Relative EphA4 mRNA expression in whole blood of naive *Cx3cr1*^*CreER*/+^*EphA4* ^+/+^ and *Cx3cr1*^*CreER*/+^*EphA4* ^*f*/*f*^ at 2 weeks and 1-month post-tamoxifen (TAM) injection. **(B)** PCR amplification of Cx3cr1-WT allele (695 bp) and Cx3cr1-mutant allele (300 bp). Heterozygous *Cx3cr1*^*CreER*/+^ shows both Cx3cr1-WT (695 bp) and Cx3cr1-mutant (300 bp) alleles. Mutant *Cx3cr1*^*CreER*/*CreER*^ shows Cx3cr1-mutant (300 bp) allele only. **(C,D)** Body weight and cortex (Ctx) mantle thickness of *EphA4* ^*f*/*f*^*, Cx3cr1*^*CreER*/*creER*^*EphA4* ^+/+^, *Cx3cr1*^*CreER*/+^*EphA4* ^+/+^, and *Cx3cr1*^*CreER*/+^*EphA4* ^*f*/*f*^ mice. ns, nonsignificant difference was observed between different groups. **(E–H)** Representative images for the contralateral cortex of Nissl-stained coronal sections taken at 4× magnification. *N* = 5–13, ns, nonsignificant difference was observed between different groups.

Supplementary Figure 4 Blood-brain barrier (BBB) permeability was measured at 3 dpi using tail vein injection of Evan's blue dye. The absorbance of Evan's blue in the ipsilateral hemispheres was measured at 610 nm and compared to the contralateral of each group. *N* = 5–6, ^*^*p* < 0.05, compared to the corresponding contralateral.
