# Supplementary figures and images for: Conditional Deletion of EphA4 on Cx3cr1-Expressing Microglia Fails to Influence Histopathological Outcome and Blood Brain Barrier Disruption Following Brain Injury
Source: Front Mol Neurosci. 2021 Sep 24;14:747770. doi: 10.3389/fnmol.2021.747770 (PMC8497746; doi:10.3389/fnmol.2021.747770)

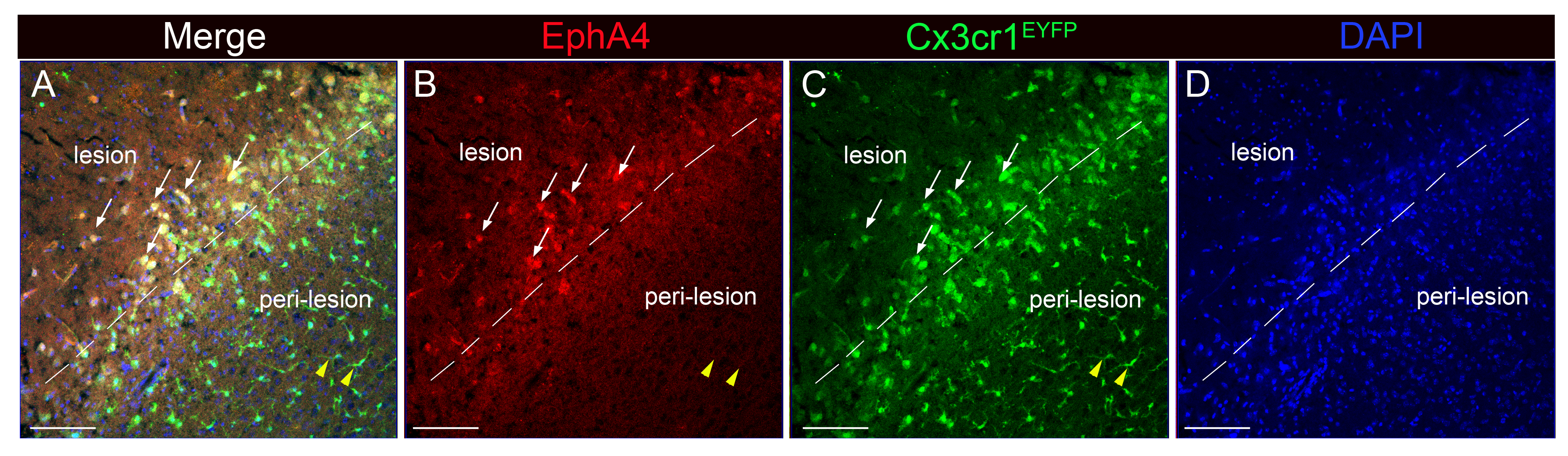

Supplement: Supplementary Figure 1 — EphA4 expression on amoeboid not ramified Cx3cr1+ cells microglia in the peri-lesion cortex. (A–D) Representative confocal images for immunohistochemical analysis for EphA4 (red), EYFP in Cx3cr1-expressing cells (green), and DAPI (blue) at 3 dpi in the ipsilateral cortex of Cx3cr1CreER/+mice. CCI injury increased EphA4 expression in amoeboid cx3cr1+ cells (white arrows) in the lesion and peri-lesion. EphA4 is not observed on ramified microglia (yellow arrowhead). Scale bar = 100 μm. [file Image_1.TIF]

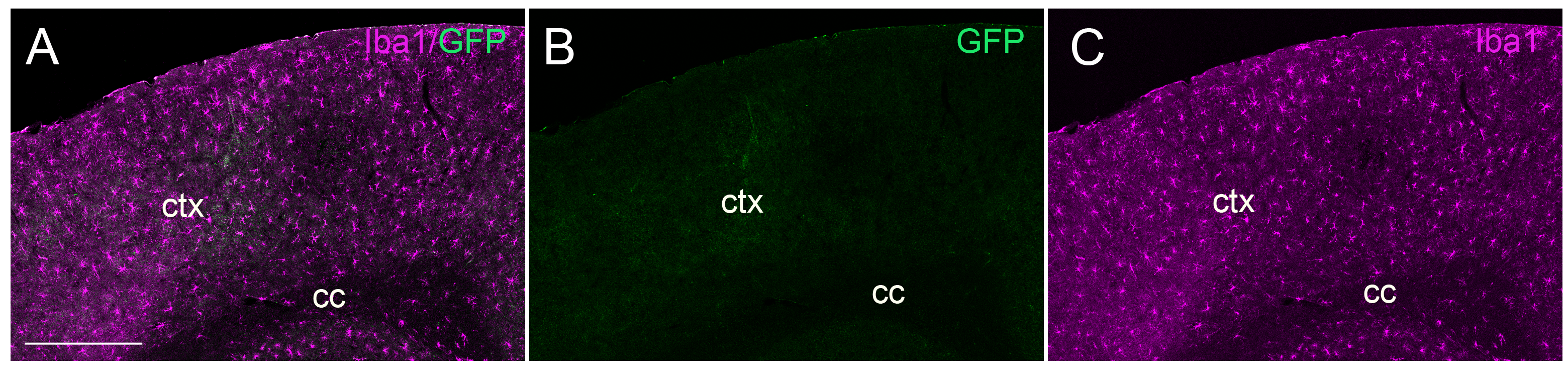

Supplement: Supplementary Figure 2 — Confocal image analysis of the contralateral cortical hemisphere at 3 dpi in GFP-adoptive transfer mice. (A) Merged confocal image max z-projection of Iba1 and GFP in chimeric mice at 3 days post-CCI injury. Individual channel images of GFP (B) and Iba1 (C). Scale bar 500 μm. ctx-cortex; cc, corpus callosum. [file Image_2.TIF]

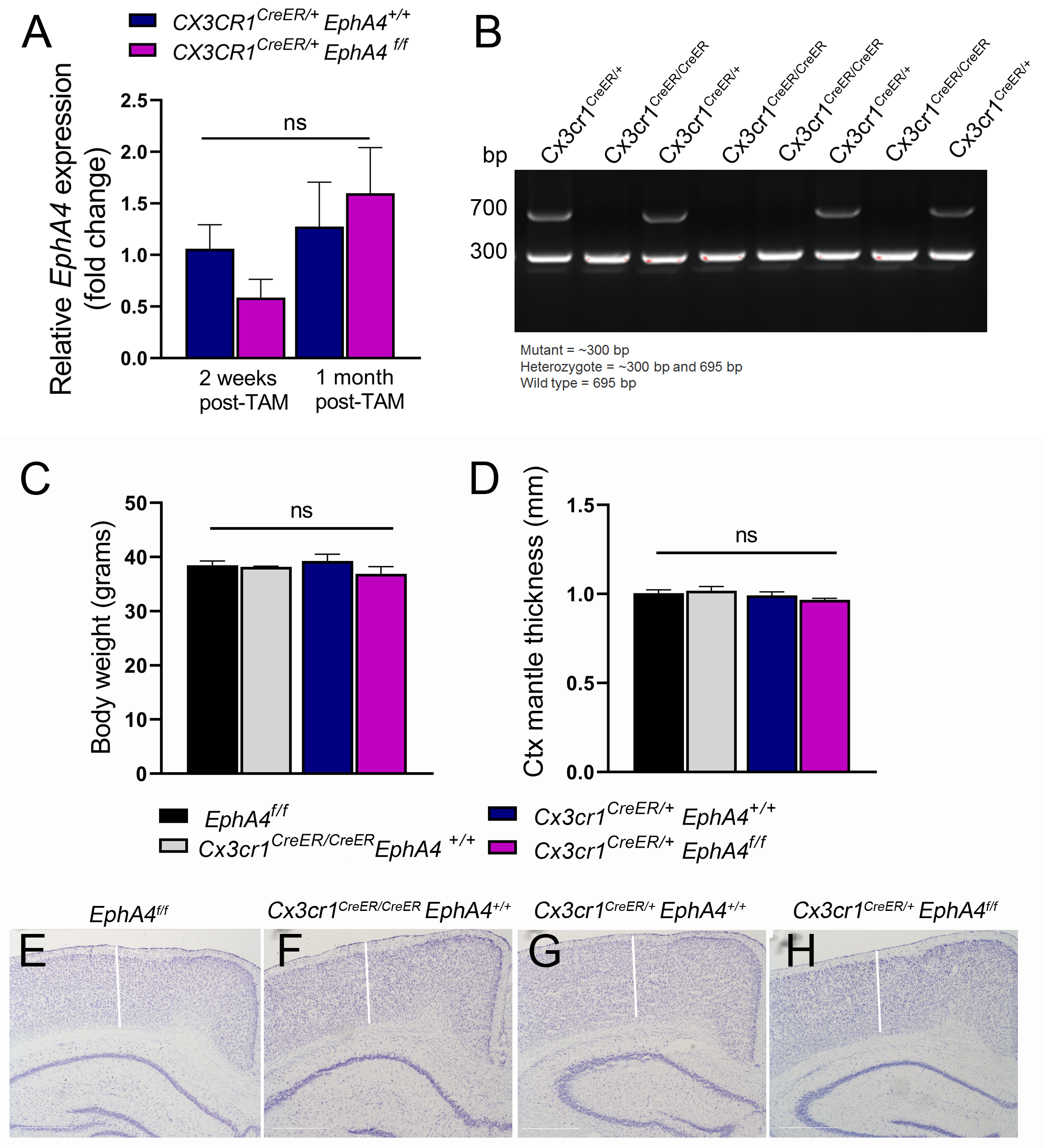

Supplement: Supplementary Figure 3 — Whole blood EphA4 expression and Cx3cr1CreER mice genotype, body weight, and cortical mantle thickness. (A) Relative EphA4 mRNA expression in whole blood of naive Cx3cr1CreER/+EphA4 +/+ and Cx3cr1CreER/+EphA4 f/f at 2 weeks and 1-month post-tamoxifen (TAM) injection. (B) PCR amplification of Cx3cr1-WT allele (695 bp) and Cx3cr1-mutant allele (300 bp). Heterozygous Cx3cr1CreER/+ shows both Cx3cr1-WT (695 bp) and Cx3cr1-mutant (300 bp) alleles. Mutant Cx3cr1CreER/CreER shows Cx3cr1-mutant (300 bp) allele only. (C,D) Body weight and cortex (Ctx) mantle thickness of EphA4 f/f, Cx3cr1CreER/creEREphA4 +/+, Cx3cr1CreER/+EphA4 +/+, and Cx3cr1CreER/+EphA4 f/f mice. ns, nonsignificant difference was observed between different groups. (E–H) Representative images for the contralateral cortex of Nissl-stained coronal sections taken at 4× magnification. N = 5–13, ns, nonsignificant difference was observed between different groups. [file Image_3.TIF]

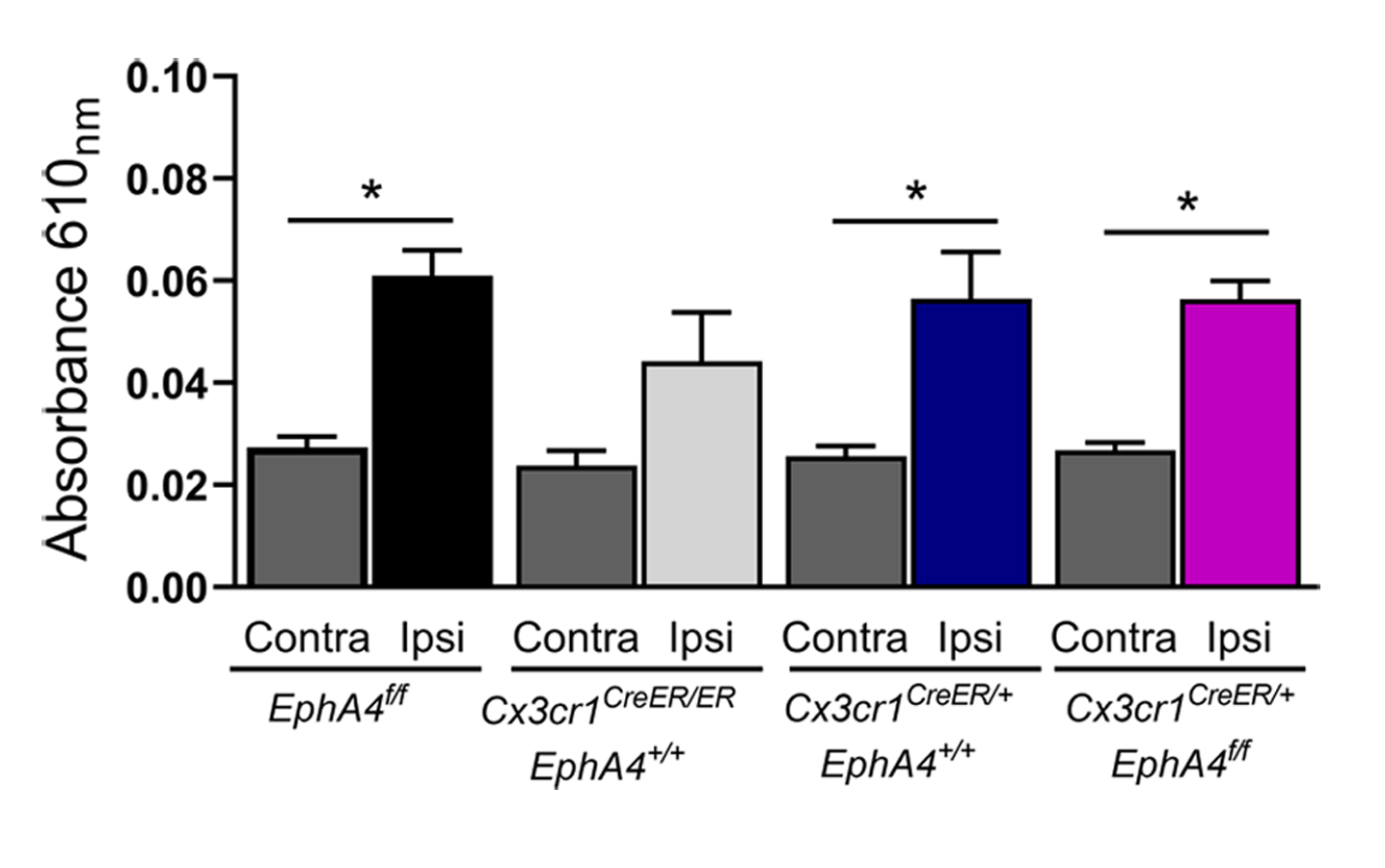

Supplement: Supplementary Figure 4 — Blood-brain barrier (BBB) permeability was measured at 3 dpi using tail vein injection of Evan's blue dye. The absorbance of Evan's blue in the ipsilateral hemispheres was measured at 610 nm and compared to the contralateral of each group. N = 5–6, *p < 0.05, compared to the corresponding contralateral. [file Image_4.TIF]
